# Effect of administration of vitamin D3 alone or combined with donepezil on the memory of adult and aged ovariectomized rat

**DOI:** 10.1002/alz.091739

**Published:** 2025-01-03

**Authors:** Lucineia Danielski, Eduarda Behenck Medeiros, Gabriel Casagrande Zabot, Adrielly Vargas, Gabriela Piovesan Fenilli, Laura Ceolin De Jesus, Gustavo De Bem Silveira, Vijayasree V Giridharan, Tatiana Barichello, Josiane Budni

**Affiliations:** ^1^ The University of Texas Health Science Center at Houston, Houston, TX USA; ^2^ Universidade do Extremo Sul Catarinense, Criciuma, SC Brazil; ^3^ University of Southern Santa Catarina (UNESC), Criciuma, SC Brazil; ^4^ Laboratory of Experimental Neurology, Graduate Program in Health Sciences (PPGCS), University of Southern Santa Catarina (UNESC), Criciuma, SC Brazil

## Abstract

**Background:**

The increasing prevalence of neurodegenerative diseases, particularly among women post‐menopause, is linked to the decline in 17 β estradiol (E2). Vitamin D deficiency, common in older individuals, exacerbates this risk due to its anti‐inflammatory and neuroprotective properties. Hypovitaminosis D is associated with age‐related conditions, including cognitive decline. Donepezil, an acetylcholinesterase inhibitor used for Alzheimer’s dementia, targets cholinergic dysfunction in normal aging and prodromal states of this disease, suggesting a potential intervention for early disease modification.

**Method:**

Adult female Wistar rats aged 60 or 120 days were used, submitted to ovariectomy (OVX) for 1, 4, or 8 months, supplemented with vit D, at doses of 42 and 420 IU/kg, or water or combined with DONE (1 mg/kg) orally by gavage for 21 days. The open field test (OFT) and Y‐maze test were performed.

**Result:**

Ovariectomized rats at 60 and 120 days of age exhibit deficits in short‐term spatial reference memory, as evidenced by performance in the Y‐maze task. In the OFT, 60‐day‐old rats subjected to OVX for four months displayed impairment, a trend also observed in rats of the same age undergoing OVX for eight months, both in the control and OVX groups. Notably, 120‐day‐old rats subjected to OVX for 4 and 8 months failed to learn, along with their respective control groups. However, the majority of interventions successfully reversed these memory impairments, with particular efficacy observed in treatments involving vitamin D at 42 IU/kg and the combination of vitamin D with DONE.

**Conclusion:**

Memory impairment occurs as a result of ovariectomy (OVX). This effect persists with aging, and vit D, either alone or in combination with DONE, emerges as a significant alternative for reversing or mitigating this cognitive decline.

